# The role of the multidisciplinary team in the management of deep infiltrating endometriosis

**DOI:** 10.1186/s10397-017-1018-0

**Published:** 2017-08-15

**Authors:** Lilian Ugwumadu, Rima Chakrabarti, Elaine Williams-Brown, John Rendle, Ian Swift, Babbin John, Heather Allen-Coward, Emmanuel Ofuasia

**Affiliations:** Croydon Endometriosis Centre, Croydon University Hospital, 530 London Road, Croydon, CR7 7YE UK

**Keywords:** Multidisciplinary team, Multidisciplinary care, Multidisciplinary meetings, Deep infiltrating endometriosis, Rectovaginal endometriosis

## Abstract

The multidisciplinary team (MDT) is considered good practice in the management of chronic conditions and is now a well-established part of clinical care in the NHS. There has been a recent drive to have MDTs in the management of women with severe endometriosis requiring complex surgery as a result of recommendations from the European Society for Human Reproduction and Embryology (ESHRE) and British Society for Gynaecological Endoscopy (BSGE). The multidisciplinary approach to the management of patients with endometriosis leads to better results in patient outcomes; however, there are potentially a number of barriers to its implementation and maintenance. This paper aims to review the potential benefits, disadvantages and barriers of the multidisciplinary team in the management of severe endometriosis.

## Introduction

Endometriosis is a common non-malignant multi-organ disease characterised by the presence of endometrial glands and stroma outside the uterus. Three clinical presentations of this condition have been described: peritoneal endometriosis, ovarian endometrioma and deep infiltrating endometriosis [1]. Deep infiltrating endometriosis (DIE) is the most aggressive form, defined as endometriosis located more than 5 mm beneath the peritoneal surface [2]. It affects the bowel and urinary tract in 5–40% and 1–4% of women with pelvic endometriosis respectively [3, 4]. When the bowel or urinary tract is involved, a combined approach with the colorectal surgeon, urologist and gynaecological surgeon is mandatory. Due to the complexity of this condition, there is greater demand on healthcare services to provide high-quality multidisciplinary care across related specialties for women with severe endometriosis. In 1995, the Calman-Hine report outlined reforms of the UK’s cancer services with the aim of reducing inequalities and improving clinical outcomes in NHS cancer care. Its main recommendation which was endorsed by the UK Department of Health was the use of multidisciplinary teams (MDT) as the core model for managing chronic conditions which is now an established part of NHS clinical care and service provisions [5]. This has also been highlighted in the European Society for Human Reproduction and Embryology (ESHRE) Guideline on the Diagnosis and Management of Endometriosis, which emphasised the complexity of the management of deep infiltrating endometriosis and the need to refer to tertiary centres with the appropriate expertise to offer all available treatments in a multi-disciplinary approach [6]. The British Society for Gynaecological Endoscopy has also established criteria for these centres carrying out complex endometriosis surgery before accreditation. One of the criteria includes working in a multi-disciplinary team with a named colorectal surgeon and nurse specialist [7].

## Methods

This aim of this paper is to evaluate the role, benefits, and drawback of multidisciplinary team management of women with deep infiltrating endometriosis. A literature search was performed using the following databases: PubMed, Medline, Ovid and Cochrane for English-language articles published from 1987 till date. The search terms used were various combinations of “multidisciplinary team”, “multidisciplinary approach”, “multidisciplinary care”, “multidisciplinary treatment”, “multidisciplinary meetings”, and deep infiltrating endometriosis. All papers and references were reviewed by the authors and relevant papers identified.

## Benefits

Multidisciplinary team work involves coordinated efforts between specialists with expertise in their disciplines in the management of a patient. These MDT meetings ensure higher quality decision-making, reduced incidence of questionable practices, standardised patient care and improved outcomes [8–10]. Endometriosis is a chronic condition and an integrated approach involving a multidisciplinary team is essential in optimising patient management. It ensures that a full range of therapeutic options are considered early so patients receive appropriate and timely treatments. The MDT led by an experienced gynaecological surgeon working together with a urologist, colorectal surgeon, specialist nurse, specialist gynaecology radiologist, pain specialist, counsellors/psychologist and patient support organisations is essential in managing complex cases [11]. They all play an important role in providing adequate treatment as well as increasing the likelihood of providing consistent, evidence based and cost effective care [12]. Patient support groups and organisations work closely and collaborate with endometriosis specialists, researchers and policy makers to increase awareness of endometriosis and drive research forward. Women also benefit from these support groups as they can share the emotional aspect of this disease, effects on their lives and families and coping strategies.

## Preoperative work-up

Preoperative work-up is important in planning a multidisciplinary surgical treatment. Reliably detecting deep infiltrating endometriosis especially in posterior compartment endometriosis could inform surgeons of the need for bowel preparation before surgery and a colorectal surgeon presence at the time of surgery. For the evaluation of bowel endometriosis, physical examination has a limited capacity to diagnose deep infiltrating endometriosis [13]. Several imaging modalities have been used to evaluate deep infiltrating endometriosis in the preoperative setting including transvaginal ultrasound and MRI of the pelvis [14]. Transvaginal ultrasound is the most studied imaging technique for deep infiltrating endometriosis, showing a pooled estimate of sensitivities and specificities of 91 and 98%, respectively [15]. Transvaginal ultrasound is operator dependent with higher accuracy obtained when performed by more experienced operators. In our practice, transvaginal ultrasound scan is recommended and performed by an experienced gynaecologist for the initial assessment of patients with suspected endometriosis and it may be useful to triage patients appropriately.

Currently, MRI is not routinely recommended in women with suspected endometriosis but could be particularly useful in detecting rectovaginal and bowel endometriosis [16–18]. Abrao et al. [19] found that MRI had a sensitivity and specificity of 83 and 98% and a recent Cochrane review [16] showed a sensitivity and specificity of 79 and 94% respectively for rectovaginal endometriosis.

A recent systematic review on ureteral endometriosis found that abdominopelvic ultrasound and/or MRI or CT-scan were routinely performed in the initial evaluation. In some studies cystoscopy was also performed when bladder infiltration was suspected [20].

These investigations aims to (1) determine disease location; (2) extent of the disease; (3) planning multidisciplinary team meetings; (4) discuss postoperative care and complications. In our referral centre, all patients with suspected DIE or rectovaginal endometriosis are discussed in our monthly multidisciplinary team meeting attended by the gynaecologist, radiologist, urologist, colorectal surgeon, and the endometriosis nurse specialist where the patient’s history, clinical examination findings and preferences are discussed, images reviewed and a recommendation is made.

## Operative treatment

Laparoscopy is the gold standard used to diagnose and to classify endometriosis [21]. The aim of endometriosis surgery is to reduce pain, reoccurrence rate, and improve fertility without compromising ovarian function. With this in mind, a multidisciplinary surgical treatment approach involving the gynaecologist, urologist and colorectal surgeon with complete excision of all endometriotic lesions is paramount to achieve better long-term outcomes [6]. Observational studies have shown that laparotomy and laparoscopy are equally effective in the treatment of endometriosis-associated pain [22]. However, laparoscopy is preferred to laparotomy because it is associated with a better postoperative recovery, shorter hospital stay, and better cosmesis [23]. Women with deep infiltrating endometriosis should be managed in a tertiary referral centre that offers advanced laparoscopic treatment in a multidisciplinary context [24]. We perform a four-step surgical procedure for these women: (1) the urologist performs a cystoscopy, inspects the bladder wall and inserts ureteric stents; (2) the gynaecologist excises all endometriosis to restore normal pelvic anatomy; (3) the urologist excises any bladder endometriosis; lastly (4) the colorectal surgeon excises bowel disease. However, if deep infiltrating endometriosis is found incidentally during laparoscopy, we will only perform what was agreed and documented on the consent form. We will inform the patient of the laparoscopy findings, discuss treatment options allowing her to make an informed decision. If the indication for laparoscopy is to manage a life threatening condition such as a ruptured ectopic pregnancy then it is in the best interest of the patient to do what is required surgically to allow adequate access to remove the ectopic pregnancy.

## Benefits to the clinician

Healthcare professionals also benefit from the multidisciplinary approach for the management of patients. Several studies have shown that it provides a framework for the understanding of the disease process thereby enabling better decision making and providing support for more complex cases [25]. Moreover, greater job satisfaction and psychological wellbeing has being demonstrated by engineering a team approach [8]. Clinicians working together in a MDT learn from each other across disciplines through active discussions, review of cases and how combined treatments can improve patient outcomes. Collaborative research is also encouraged within MDTs, which promotes greater participations in clinical trials, which helps to improve the understanding of this condition, diagnosis and ensure effective treatment options [25]. It also offers educational opportunities for trainees and medical students who can gain greater insight into the importance of multidisciplinary team work in the management of patients with chronic conditions.

## Disadvantages

One of the disadvantages of MDT discussions is the lack of patient involvement since patients are not present at these meetings. If patient preferences or social circumstances are not taken into account, team decisions may be inappropriate or rejected. However, patient attendance at these meetings may not be beneficial because of their limited understanding of medical terminology, which may restrict the free flow of information and in addition lead to ineffective input from patients. This may be potentially overcome by the use of questionnaires in the clinic which could guide the MDT in recommending a particular treatment plan best suited to the patient. The effective functioning of an MDT requires constructive input from all team members. A lack of clear roles, objectives and also enthusiasm from its members can hinder the development of constructive management. This does not only have implications for patient care and safety but also has medicolegal implications. All professionals who attend team meetings have a duty of care for decisions made [26]. Clear documentation is important to improve communication between team members and also with the patient. In our centre, the gynaecologist is the primary clinician who takes responsibility for patient care with input from other related specialties. All recommendations following MDT meetings are documented in patients’ notes for reference.

## Barriers

Although it has been established that multidisciplinary team management improves patient outcome, there are a number of barriers that prevent the full realisation of these benefits. Such barriers include cost, time constraints, and poor interprofessional relationships. The estimated total monthly cost of gynaecological cancer MDTs in the UK is £101,880 [27]. This prompts the debate of the cost-effectiveness of MDTs when used for routine benign cases. The cost should be balanced with the cost of reduced economic productivity from patients with severe endometriosis. The WERF EndoCost study has shown that the costs of productivity loss of €6298 per woman were double the health care costs of €3113 per woman suffering from endometriosis-associated symptoms and treated in referral centres [12]. In 2005, the UK Endometriosis All Party Parliamentary Group (EAPPG) carried out a survey on pain and quality of life. They showed that 78% of symptomatic women with endometriosis lose a mean of 5.3 days of work a month because of their symptoms, with a potential cost of €30 billion across Europe [28]. Additionally, there is a diagnostic delay of over 8 years with 65% of women with endometriosis initially misdiagnosed and almost 50% having to see five doctors or more before a correct diagnosis is made. This is likely to increase the cost to the woman if she is unable to work and the cost to the healthcare system [28]. The indirect cost of infertility treatment, drugs and surgery in women with chronic pelvic pain is estimated at £24 million in the UK [29].

Therefore, early referral to a tertiary centre where the appropriate skills and expertise exist to make the correct diagnosis and implement effective management of endometriosis will significantly reduce time to diagnosis and costs.

As a minimum, time required to facilitate MDT meetings and treatment plans should be included in job plans to allow an effective high quality service. Poor interprofessional relationship can also affect teamwork and hinder shared responsibility.

## Conclusions

Although informal discussions already exist in many hospitals, a formalised multidisciplinary preoperative work up and surgical treatment in an endometriosis referral centre is necessary to plan patients counselling and treatment plan implementation which assures improved outcomes. This should be carried out in collaboration with the MDT including a gynaecologist, urologist, colorectal surgeon, specialist nurse, radiologist, pain specialist, counsellors/psychologist and patient support organisations. The success of MDT’s in cancer care should encourage its uptake in benign but chronic conditions such as endometriosis. In future, research should focus on the effect of MDT’s on patient long-term outcomes, cost effectiveness and perception amongst clinicians.
